# Association of maternal levothyroxine use during pregnancy with offspring birth and neurodevelopmental outcomes: a population-based cohort study

**DOI:** 10.1186/s12916-022-02586-9

**Published:** 2022-11-08

**Authors:** Grace Mengqin Ge, Edmund C. L. Cheung, Kenneth K. C. Man, Patrick Ip, Wing Cheong Leung, Gloria H. Y. Li, Annie W. C. Kung, Ching-Lung Cheung, Ian C. K. Wong

**Affiliations:** 1 Centre for Safe Medication Practice and Research, Department of Pharmacology and Pharmacy, Li Ka Shing Faculty of Medicine, The University of Hong Kong, Hong Kong Special Administrative Region, China; 2 Research Department of Practice and Policy, UCL, School of Pharmacy, London, UK; 3 Department of Paediatrics and Adolescent Medicine, Li Ka Shing Faculty of Medicine, The University of Hong Kong, Hong Kong Special Administrative Region, China; 4 Department of Obstetrics and Gynecology, Kwong Wah Hospital, Hong Kong Special Administrative Region, China; 5 Department of Health Technology and Informatics, Faculty of Health and Social Sciences, The Hong Kong Polytechnic University, Hong Kong Special Administrative Region, China; 6 Department of Medicine, Li Ka Shing Faculty of Medicine, The University of Hong Kong, Hong Kong Special Administrative Region, China; 7 Aston Pharmacy School, Aston University, Birmingham, B4 7ET UK

**Keywords:** attention-deficit/hyperactivity disorder, autism spectrum disorder, birth outcomes, levothyroxine, pregnancy, maternal, offspring

## Abstract

**Background:**

The influence of maternal levothyroxine treatment during pregnancy remains unclear. This study aimed to evaluate the associations of maternal levothyroxine treatment during pregnancy with the birth and neurodevelopmental outcomes in offspring.

**Methods:**

This population-based cohort study was conducted among pregnant women using the Hong Kong Clinical Data Analysis and Reporting System. Mother-child pairs in Hong Kong from 2001 to 2015 were included and children were followed up till 2020. We defined the exposure group as mothers who were exposed to levothyroxine during pregnancy. Preterm birth and small for gestational age (SGA) were included as birth outcomes. Attention-deficit/hyperactivity disorder (ADHD) and autism spectrum disorder (ASD) were included as neurodevelopmental outcomes. Odds ratios (OR) or hazard ratios (HRs) with a 95% confidence interval (CI) were evaluated to assess the association of gestational levothyroxine use with offspring birth and neurodevelopmental outcomes respectively, using propensity score fine-stratification weighting and a Cox proportional hazards regression model.

**Results:**

Among 422,156 mother-child pairs, 2125 children were born from mothers exposed to levothyroxine during pregnancy. A significantly increased risk of preterm birth was observed in children with maternal levothyroxine exposure during pregnancy, when compared to mothers who had no history of thyroid-related diagnoses or prescriptions (weighted OR [wOR]: 1.22, 95% CI: 1.07, 1.39). Similarly, an increased risk of preterm birth was found among children of gestational levothyroxine users, when compared to children of mothers who had used levothyroxine before but stopped during pregnancy (wOR: 2.16, 95% CI: 1.09, 4.25). Sensitivity analysis, by excluding mothers exposed to psychotropic or antiepileptic medications before or during pregnancy, also indicated a similar increased risk of preterm birth regarding the gestational use of levothyroxine (wOR: 1.26, 95% CI: 1.10, 1.45). No significant association was observed for the risk of SGA, ADHD, and ASD.

**Conclusions:**

There is no evidence that gestational use of levothyroxine is associated with SGA, ADHD, or ASD in offspring. Gestational levothyroxine treatment is associated with a higher risk of preterm birth. Such risk might be confounded by the underlying maternal thyroid disease itself, however, we cannot completely exclude the possible effect of gestational L-T4 treatment on offspring preterm birth. Our findings provided support to the current guidelines on the cautious use of levothyroxine treatment during pregnancy.

**Supplementary Information:**

The online version contains supplementary material available at 10.1186/s12916-022-02586-9.

## Background

Thyroid diseases are the second most common endocrine disorder in pregnancy, which are broadly categorized into hyperthyroidism and hypothyroidism, with a prevalence of around 0.2% and 2.5%, respectively [1–6]. As thyroid physiology is distinctly modified during pregnancy [7], the proper transferal of maternal thyroid hormones to the developing fetus is critical, especially during the early stages of gestation. The growth of the fetus at these stages is completely dependent on the maternal thyroid source as the fetal thyroid is not fully developed [8]. Thyroid hormone is particularly important for fetal brain development [8, 9], thus dysregulation of maternal thyroid hormone levels may lead to the development of neurodevelopmental disorders in the offspring. Maternal hypothyroidism was reported to be associated with various birth and fetal neurodevelopmental problems including preterm birth [10, 11], reduced fetal growth [11], attention-deficit/hyperactivity disorder (ADHD) [12], and autism spectrum disorder (ASD) [13].

Though previous studies have suggested the association of maternal hypothyroidism with increased risk of neurodevelopmental disorders of the offspring [14], little is known about the influence of maternal levothyroxine (L-T4) treatment during pregnancy. Results from a few randomized trials suggested that L-T4 treatment of subclinical hypothyroidism or hypothyroxinemia did not affect pregnancy outcomes or cognitive functions in offspring [15–17]. Analysis of the data generated from a trial of universal screening for thyroid dysfunction reported that women treated with L-T4 were less likely to experience adverse maternal and fetal outcomes [18]. However, the effect of L-T4 treatment in mothers with autoimmune thyroid disorders was inconclusive, with a beneficial effect observed in prospective studies [19, 20], while a null effect was observed in a randomized trial [21]. Importantly, most of the studies focused on the effect of maternal L-T4 treatment on pregnancy outcomes but none of them was designed to investigate long-term neurodevelopmental disorders in the offspring.

Considering the inconsistency and uncertainty of the findings in previous studies, this study aimed to estimate the associations of maternal L-T4 treatment during pregnancy with birth and neurodevelopmental outcomes in offspring using a large real-world cohort of pregnant women in Hong Kong.

## Methods

### Data source and study design

This was a territory-wide retrospective cohort study using data from the Hong Kong Clinical Data Analysis and Reporting System (CDARS), which is operated by the Hospital Authority (HA) that manages all public hospitals and the associated ambulatory clinics in Hong Kong. CDARS contains electronic medical records of over 7.5 million Hong Kong residents, including their demographics, diagnosis, and prescription records. The database has been used for various high-quality epidemiological studies [22–25], including studies that examined the safety of prenatal medication use on birth/neurodevelopmental complications [26–28].

### Study population

This study included mother-child pairs in Hong Kong from 1st January 2001 to 31st December 2015. The children were followed up till 31st December 2020, resulting in at least 5 years of follow-up time. We defined a valid mother-child linkage as an exact match of mother and child identification numbers, delivery date, and delivery hospital. Given that the mother-child linkage is created deterministically by the HA for clinical management where the mother and the child records are linked permanently immediately after delivery, this linkage is considered highly accurate [22]. All the outcomes are associated with live birth with the delivery date as the index date; therefore we excluded perinatal death cases or pregnancy episodes of incomplete birth information, such as missing gestational age and birth weight.

### Exposure and comparison groups

We defined L-T4 users as mothers with ≥2 dispensing records for thyroid hormone treatment (listed in chapter 6.2.1 of the British National Formulary [BNF]) before giving birth. In Hong Kong, anti-thyroid drugs are the first-line therapy for hyperthyroidism treatment, while thyroidectomy or radioactive iodine (RAI) therapy are the alternatives when anti-thyroid drug treatments fail. Patients receiving thyroidectomy/RAI often require the prescription of L-T4 afterwards when overt hypothyroidism develops. Therefore, we excluded mothers exposed to anti-thyroid drugs, thyroidectomy, or RAI during the study period to make sure that mothers in the exposure group have relatively similar thyroid status and to avoid potential misclassification.

We defined the pregnancy period as the period between the last menstrual period (LMP) and the date of delivery (Additional file 1: Fig. S1). The gestational week was directly recorded by healthcare professionals and the LMP was defined by the date of delivery minus the gestational week at delivery. We classified the children based on maternal L-T4 exposure status. In our main analysis, we compared children with mothers who received L-T4 treatment during pregnancy (*gestational users*) to those with euthyroid mothers who have never been exposed to L-T4 and had no history of thyroid-related disorders (*euthyroid control*). Thyroid-related disorders are defined by the International Classification of Diseases, 9th Revision, Clinical Modification (ICD-9-CM) codes 242-244 in the mother’s longitudinal medical records. The look-back period started from: (1) the beginning of the electronic health record set up in 1995 or (2) the birth of the mother or (3) the first time the mother used HA services. For *gestational users*, we calculated the median of gestational weeks that mothers started their L-T4 treatments in order to compare with previously published studies.

### Main outcomes and follow-up

Our study examined birth outcomes including preterm birth and small for gestational age (SGA), and neurodevelopmental outcomes including ADHD and ASD. Preterm birth was defined as the delivery of babies before 37 gestational weeks. SGA was defined as offspring whose birth weight was <2 standard deviations (SD) below the mean of the same gestational age. ADHD was defined using an ICD-9-CM record of 314 or a prescription for methylphenidate or atomoxetine. ASD was defined using an ICD-9-CM record of 299. CDARS contains hospital diagnoses from specialists, so the positive predictive value of the neurodevelopmental outcomes is high. The database has previously been used to study ADHD and ASD in children and has been accepted to be a valid database [26–30].

For neurodevelopmental outcomes, the follow-up in children started on the date of delivery and ended on the date of occurrence of the outcome, date of death, or 31 December 2020, whichever came first.

### Covariates

Data on maternal comorbidities were obtained from medical records in CDARS. Covariates for adjustment were based on previous literature [31–34], including maternal age at delivery, parity, calendar year at delivery, birth hospital, and underlying medical conditions in mothers including hypertension, epilepsy, psychiatric disorders, gestational diabetes, and pre-existing diabetes (ICD-9-CM codes for the covariates are shown in the Additional file 2: Table S1). Maternal underlying medical conditions were defined using the diagnosis history recorded by physicians before mothers’ deliveries.

### Statistical analysis

We performed Propensity Score (PS) fine-stratification weighting to address the differences in baseline covariates. PS methods have advantages over conventional covariate adjustment and have been increasingly applied to adjust for confounding in observational studies [35]. PS fine-stratification weighting was selected in this study as it performs greater precise estimates than other PS methods at low exposure prevalence [36, 37]. This approach allows us to create fine strata and to calculate weights for both exposed and reference patients in all strata based on the total number of patients within each stratum [36]. Strata with no exposed or reference patients were dropped out before weight calculation. In this study, 150 equally sized strata were created based on the PS distribution of the cohort. We used the “average effect of the treatment on the treated” for PS fine-stratification in this study, as the mothers’ characteristics are more likely to determine the treatment received [38]. We calculated the standardized mean differences (SMD) to examine the covariates balance between comparison groups. An SMD value of less than 0.1 for covariates was considered as well-balanced [37].

We presented proportions for all the outcomes among different comparison groups and PS-weighted cumulative incidence of the neurodevelopmental diagnoses. We presented PS-weighted odds ratios (wORs) with 95% confidence interval (CI) using logistic regression models to assess the association of maternal L-T4 use with birth outcomes of the offspring, and PS-weighted hazard ratios (wHRs) with 95% CI using Cox proportional hazard regression models to assess the association of maternal L-T4 use with neurodevelopment outcomes in offspring. Robust standard errors were used to adjust for the clustering of individuals bound by the same biological mother.

Additional analyses were conducted to test the validity and robustness of the initial analyses. First, we identified children with mothers exposed to L-T4 before pregnancy but stopped the treatment when pregnant (*pre-pregnancy users*) and calculated the median time from *pre-pregnancy users*’ last prescription of L-T4 till their LMP. We adapted the analytical strategy from a previous study on maternal drug exposure and neurodevelopmental disorders to develop our sensitivity analysis by comparing *gestational users* to *pre-pregnancy users* [28]. If there is an increased risk in *gestational users* compared to *euthyroid control*, it could be due to the use of levothyroxine and/or presence of hypothyroidism. If there is no difference or a decreased risk in *gestational users* when compared to *pre-pregnancy users*, then it suggests that the association may be due to confounding by maternal hypothyroidism. Second, since maternal exposure to psychotropics such as antidepressants, antipsychotics, lithium, and antiepileptic medications may increase the risk of giving birth to a child with birth and neurodevelopmental problems [39, 40], we further excluded mothers exposed to psychotropic or antiepileptic medications before or during pregnancy. Third, to estimate sex-specific effects, we did a subgroup analysis stratifying by the offspring’s sex. Fourth, in the main analysis, we used the SGA definition recommended by a consensus statement of the International Societies of Pediatric Endocrinology and the Growth Hormone Research Society [41]. In this sensitivity analysis we applied different SGA definitions: the lowest 3rd or 5th percentile of the gestational age-specific birth weight within the cohort of live births. Fifth, we conducted a sensitivity analysis considering the severity of preterm birth. In our main analysis, our preterm birth definition refers to moderate to late preterm birth (less than 37 gestational weeks). We applied other cut-offs to identify severe preterm birth, including less than 33 weeks as very preterm birth and less than 28 gestational weeks as extremely preterm birth. Given that pre-eclampsia could be a risk factor for preterm birth [42], we conducted additional post hoc sensitivity analysis by adjusting maternal pre-eclampsia using PS fine stratification for different cut-offs of preterm births. Sixth, as most cases of ADHD can only be diagnosed after the age of 5 to 6 years old, children delivered in the year after 2014 may not have follow-up long enough to accurately detect ADHD risk. Therefore, when ADHD was evaluated as the outcome, we repeated the analyses only in children born before the year 2014 to make sure there were at least 7 years of follow-up. Seventh, the guidelines of the American Thyroid Association (ATA) revised their upper limit for thyroid-stimulating hormone (TSH) during pregnancy in 2011 [4]. The upper limit of 2.5 mIU/L for serum TSH in the 1st trimester has become internationally accepted after the revision. Therefore, we repeated the main analysis in subgroups of before and after the year 2011. We also conducted an interaction analysis to see if there is a significant interaction before and after the 2011 ATA guideline change on the observed estimates.

Finally, we conducted post hoc sensitivity analysis considering the cumulative dose of L-T4 during pregnancy and the length of time mothers used L-T4 before pregnancy within the gestational users. We calculated the cumulative dose in each pregnancy episode of the exposed mothers, then subgrouped the gestational exposure group by their cumulative dose. We categorized less than the median cumulative dose as the low cumulative dose group and more than the median cumulative dose as the high cumulative dose group. We then conducted a subgroup analysis by additional comparisons between the low cumulative dose, high cumulative dose, and euthyroid groups. Similarly, we calculated cumulative days that mothers were using L-T4 before their LMP. We categorized less than median cumulative days as the short cumulative days group and more than median cumulative days as the long cumulative days group. We conducted a subgroup analysis by comparing the outcomes among the short cumulative days, long cumulative days, and euthyroid groups.

A significance level of 0.05 was used in all statistical analyses. All analyses were conducted using Statistical Analysis System (SAS) v9.4 (SAS Institute, Cary, NC) and R (R Core Team, 2020). The results were cross-checked for accuracy and consistency by GG and EC.

## Results

**Fig. 1:**
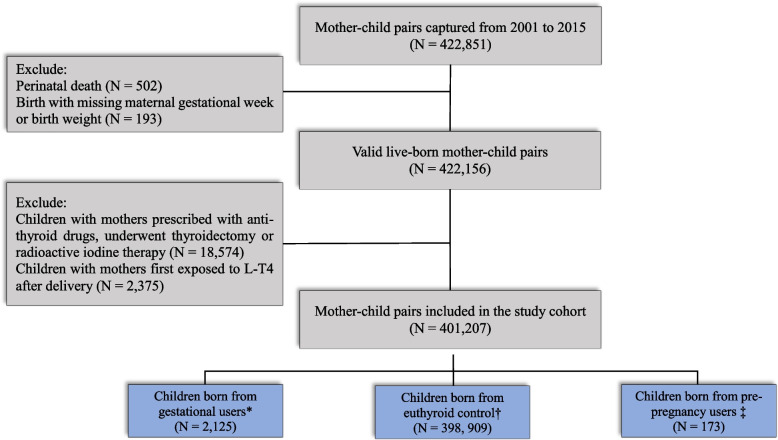
Flowchart of mother-child pairs identification. A total of 422,851 mother-child pairs were captured from 2001 to 2015. A total of 502 perinatal death cases and 193 mother-child pairs with missing gestational age and birth weight were excluded. Mothers exposed to anti-thyroid drugs, underwent thyroidectomy or radioactive iodine therapy during the study period, and mothers first exposed to L-T4 after giving birth were also excluded. The final cohort included 401,207 mother-child pairs. There were 2125 children born from mothers who received L-T4 during pregnancy, and 398,909 children born from mothers who had no history of thyroid-related diagnoses or prescriptions. There were 173 children born from mothers exposed to L-T4 before pregnancy but stopped the treatment when pregnant

The original data included 422,851 mother-child pairs from 2001 to 2015. Five hundred two perinatal death cases and 193 mother-child pairs with missing gestational age and birth weight were excluded. Then, after removing mothers exposed to anti-thyroid drugs, underwent thyroidectomy or RAI therapy during the study period, and mothers first exposed to L-T4 after giving birth, the cohort included 401,207 mother-child pairs. There were 2125 children born from mothers who received L-T4 during pregnancy, and 398,909 children born from mothers who had no history of thyroid-related diagnoses or prescriptions. A total of 173 children were born from mothers exposed to L-T4 before pregnancy but stopped the treatment when pregnant (Fig. 1).

### Descriptive statistics stratified by maternal L-T4 exposure

The percentages of preterm birth, SGA, ADHD, and ASD in children were respectively 12.05%, 1.93%, 4.00%, and 3.06% in the group of mothers exposed to L-T4 during pregnancy, and 8.39%, 1.70%, 3.80%, and 2.71% in the group of mothers who were euthyroid controls. The mean follow-up time of ADHD and ASD were respectively 10.60 (SD=4.12) and 10.60 (SD=4.20) years in children of gestational L-T4 users, and 10.75 (SD=3.87) and 10.77 (SD=3.95) years in children of euthyroid control mothers (Table 1). Children born from gestational L-T4 users had a similar probability of ADHD and ASD diagnosis when compared to children born from euthyroid control mothers (Additional file 1: Fig. S2).

**Table 1:** Characteristics of mothers and their children by maternal L-T4 exposure. Values are numbers (percentages) unless stated otherwise

Characteristics	Gestational users (***N*** = 2125)	Euthyroid control (***N*** = 398,909)
**Mother**		
**Mean (SD) maternal age at delivery (years)**	33.95 (4.61)	31.50 (5.04)
**Maternal underlying conditions:**		
***Gestational diabetes***	21 (0.99)	1047 (0.26)
***Pre-existing diabetes***	188 (8.85)	20,200 (5.06)
***Hypertension***	153 (7.20)	15,118 (3.79)
***Epilepsy***	15 (0.71)	653 (0.16)
***Psychiatric illness***	59 (2.78)	5889 (1.48)
**Parity:**		
***0***	1078 (50.73)	209,812 (52.60)
***1***	798 (37.55)	148,571 (37.24)
***2***	193 (9.08)	31,866 (7.99)
≥***3***	56 (2.64)	8660 (2.17)
**Children**		
**Outcomes:**		
***Preterm birth***	256 (12.05)	33,462 (8.39)
***Small for gestational age***	41 (1.93)	6787 (1.70)
***Attention-deficit/hyperactivity disorder***	85 (4.00)	15,152 (3.80)
***Autism spectrum disorder***	65 (3.06)	10,827 (2.71)
**Mean (SD) follow-up time (patient years):**		
***Attention-deficit/hyperactivity disorder***	10.60 (4.12)	10.75 (3.87)
***Autism Spectrum Disorder***	10.60 (4.20)	10.77 (3.95)
**Girls**	985 (46.35)	191,799 (48.08)
**Normal spontaneous delivery**	1139 (53.60)	262,537 (65.81)
**Multiple pregnancy**	136 (6.40)	12,759 (3.20)
**Birth trauma**	7 (0.33)	1532 (0.38)
**Timing of Apgar score <7:**		
***1 min***	114 (5.36)	13,691 (3.43)
***5 min***	18 (0.85)	1,658 (0.42)
**Birth weight (g):**		
***<1500***	55 (2.59)	4,788 (1.20)
***1500–2500***	228 (10.73)	31,570 (7.91)
***>2500***	1842 (86.68)	362,551 (90.89)
**Gestational weeks**		
≤***32***	73 (3.44)	7359 (1.84)
***33–36***	183 (8.61)	26,103 (6.54)
≥***37***	1869 (87.95)	365,447 (91.61)

*SD* standard deviation

### Main analysis

**Fig. 2:**
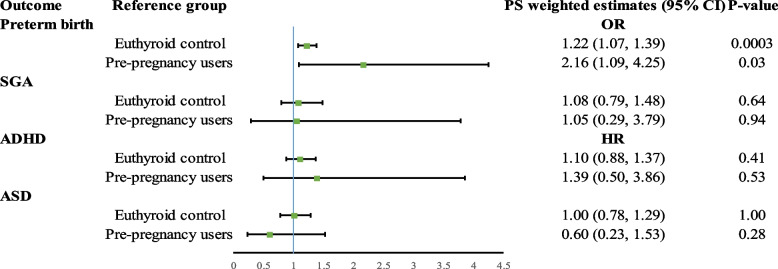
Propensity score weighted estimates from different comparison groups. A significantly increased risk of preterm birth was observed in children with maternal levothyroxine exposure during pregnancy when compared to mothers from the euthyroid control group (PS weighted OR: 1.22, 95% CI: 1.07, 1.39). An increased risk of preterm birth was also observed among children of gestational levothyroxine users when compared to children of mothers from the pre-pregnancy users group (PS weighted OR: 2.16, 95% CI: 1.09, 4.25). No significant association was observed for the risk of SGA, ADHD, and ASD

After PS weighting, all covariates were well-balanced between the groups with SMDs of less than 0.1 (Additional file 2: Table S2). Compared to children born from *euthyroid control* mothers, those born from *gestational L-T4 users* had a higher risk of preterm birth with an elevated wOR of 1.22 (95% CI: 1.07, 1.39), but not for SGA (wOR=1.08, CI: 0.79, 1.48). In terms of neurodevelopmental outcomes, no significant association was observed for the risk of ADHD and ASD between children of *gestational L-T4 users* and *euthyroid control mothers*, with a wHR of 1.10 (95% CI: 0.88, 1.37) and 1.00 (0.78, 1.29), respectively (Table 2, Fig. 2). *Gestational L-T4 users* started L-T4 treatment at a median of 18 gestational weeks (Additional file 2: Table S3).

**Table 2:** Comparison of children born from gestational L-T4 users with reference to children of euthyroid control mothers

Gestational L-T4 users (***N*** = 2125) vs euthyroid control (***N*** = 398,909)						
Outcomes	***N*** of cases in exposed (%)	***N*** of cases in unexposed (%)	Crude		PS-weighted ^a^	
			**OR (95% CI)**	***P*-value**	**OR (95% CI)**	***P*-value**
**Preterm birth**	256 (12.05)	33,462 (8.39)	1.50 (1.32, 1.71)	<0.0001	1.22 (1.07, 1.39)	0.0032
**SGA**	41 (1.93)	6787 (1.70)	1.14 (0.83, 1.55)	0.42	1.08 (0.79, 1.48)	0.64
			**HR (95% CI)**	***P*-value**	**HR (95% CI)**	***P*-value**
**ADHD**	85 (4.00)	15,152 (3.80)	1.10 (0.89, 1.36)	0.37	1.10 (0.88, 1.37)	0.41
**ASD**	65 (3.06)	10,827 (2.71)	1.15 (0.90, 1.47)	0.26	1.00 (0.78, 1.29)	1.00

*OR* odds ratio, *HR* hazard ratio, *PS* propensity score, *vs* versus, *ADHD* attention-deficit/hyperactivity disorder, *ASD* autism spectrum disorder, *SGA* small for gestational age

^a^PS-weighted model adjusted for maternal age at delivery, birth year, birth hospital, parity, maternal underlying illness before delivery including pre-existing diabetes, gestational diabetes, epilepsy, hypertension, and psychiatric conditions

### Subgroup and sensitivity analyses

All covariates were balanced after PS weighting by comparing *gestational users* to *pre-pregnancy users* (Additional file 2: Table S4). The median time from pre-pregnancy users’ last prescription of L-T4 till LMP was 768 days (Additional file 2: Fig. S3). The risk of preterm birth in children of *gestational users* was higher than in children of *pre-pregnancy users* (wHR=2.16, 95% CI: 1.09, 4.25), but no statistically significant difference was observed for SGA, ADHD, and ASD (Fig. 2; Additional file 2: Table S5).

Sensitivity analysis by restricting the analyses to a subgroup of children born from mothers without exposure to psychotropics or anti-epileptics before or during pregnancy showed a consistent pattern of results (Additional file 2: Table S6). Children born from *gestational L-T4 users* had a higher risk of preterm birth when compared to children of *euthyroid control mothers* (wOR=1.26, 95% CI: 1.10, 1.45), and a null association was observed for SGA, ADHD, and ASD.

Subgroup analysis by stratifying offspring sex suggested a similar pattern of results (Additional file 2: Table S7). Both girls and boys born from *gestational L-T4 users* had a higher risk of preterm birth when compared to girls and boys of *euthyroid control mothers* (Girls: wOR=1.23, 95% CI: 1.01, 1.50; Boys: wOR=1.20, 95% CI: 1.00, 1.43). For SGA, ADHD, and ASD, the estimates were higher in boys compared to that in girls, but no statistically significant difference was observed between children of *gestational L-T4 users* and *euthyroid control*.

Sensitivity analysis by using different SGA definitions presented similar results. No statistically significant difference was observed for SGA when comparing *gestational L-T4 users* to *euthyroid control* using either SGA definition (Additional file 2: Table S8).

For sensitivity analysis by using different cut-offs for preterm birth, we observed that *gestational L-T4 users* had a higher risk of very or extremely preterm birth compared to *euthyroid control mothers* (very: wOR=1.43, 95% CI: 1.14, 1.82; extremely: wOR=1.94, 95% CI: 1.27, 2.96). The results remained unchanged after adjusting maternal pre-eclampsia in the PS fine stratification model. (Additional file 2: Table S9).

In assessing the association of maternal L-T4 exposure during pregnancy with the risk of ADHD, subgroup analysis by restricting children born before 2014 also showed similar results (Additional file 2: Table S10). No significant difference was observed between children of *gestational L-T4 users* and *euthyroid control*.

The subgroup analysis of gestational L-T4 users before and after the year 2011 showed similar results as the main analysis (Additional file 2: Table S11). Before the year 2011, children born from *gestational L-T4 users* had a higher risk of preterm birth compared to children born from *euthyroid control* (wOR=1.21, 95% CI: 1.02, 1.45). The increased risk of preterm birth was also observed in the after 2011 group, but the estimate was marginally significant (wOR=1.21, 95% CI: 0.98, 1.49). For SGA, ADHD, or ASD, no significant difference was observed between children of gestational L-T4 users and euthyroid control. The interaction analysis suggests that the periods before and after the year 2011 did not affect the estimates in the comparison between gestational L-T4 users and euthyroid control (Additional file 2: Table S12).

The median cumulative dose in each pregnancy episode of mothers in the gestational exposed group was 13,150 mcg (Additional file 1: Fig. S4). The results in the post-hoc sensitivity analysis were essentially unchanged when the high cumulative dose and low cumulative dose groups were compared with the euthyroid group. Notably, no differences were observed when comparing the high and low dose groups directly (Additional file 2: Table S13).

The median length of time that gestational exposed mothers used L-T4 before their LMP was 843 days (Additional file 1: Fig. S5). We observed null associations in the comparison between the short cumulative days group before pregnancy and the euthyroid group for offspring birth and neurodevelopmental outcomes. However, when comparing the long cumulative days group before pregnancy to the euthyroid group, the results were similar to the main analyses. We did not observe differences when comparing short to long cumulative duration directly (Additional file 2: Table S14).

## Discussion

The present study demonstrated that maternal L-T4 use during pregnancy, compared to euthyroid control, was associated with an increased risk of preterm birth, and a higher risk of very or extremely preterm birth. A marginally elevated risk of preterm birth was also observed in our sensitivity analysis by comparing gestational users with pre-pregnancy users, who stopped L-T4 treatment before pregnancy. There could be a potential effect of gestational exposure to L-T4 on the increased risk of preterm birth in the offspring. However, we cannot exclude the possibility that the severity of maternal hypothyroidism may also play a role in the association. In our post hoc analysis, we considered the cumulative length of previous exposure to L-T4 treatment as a proxy of disease severity within gestational users, as mothers with longer cumulative exposure to treatment before pregnancy may have had a more severe hypothyroid problem. We observed an increased risk of preterm birth in the long cumulative days group compared to the euthyroid group, but no significant increased risk of preterm birth was observed when comparing the short cumulative days group to the euthyroid group. The results further supported our hypothesis that the observed increased risk of preterm birth among gestational L-T4 users compared to the euthyroid control group may also be associated with the severity of maternal hypothyroidism.

The median time from pre-pregnancy users’ last prescription of L-T4 to LMP was more than 2 years, suggesting that the majority of the pre-pregnancy users may have had better control of their thyroid status and stopped using L-T4 long before their pregnancy. This may explain the similarities between the comparisons of gestational users with pre-pregnancy and euthyroid control. Importantly, individuals who require L-T4 treatment in general may have a more severe thyroid problem, leading to their offspring having an expected higher risk of the outcomes. However, we did not observe statistically significant differences in the risk of SGA, ADHD, and ASD when comparing gestational users to euthyroid control, or when comparing gestational users to pre-pregnancy users. Thus, our findings support that there is no causal relationship between gestational exposure to L-T4 and offspring SGA, ADHD, and ASD.

### Comparison with other studies

Previous studies suggested that L-T4 overtreatment and excessive T4 may lead to infants being born preterm [43, 44], which could be one potential explanation for the observed increased risk of preterm birth observed in the comparison between gestational users and both euthyroid controls and pre-pregnancy users. Nevertheless, in this study, we observed an increased risk of preterm birth in both high and low cumulative dose groups when compared to the euthyroid group. No differences were observed between high and low cumulative dose groups. Notably, hypothyroidism has been known to be associated with an increased risk of preterm birth [45], however, to the best of our knowledge, no existing clinical trials ever examined if L-T4 treatment among hypothyroid women during pregnancy would reduce such a risk. One recently published meta-analysis reported that L-T4 treatment in pregnant women with subclinical hypothyroidism may reduce the risk of preterm birth [46]. However, this meta-analysis focused on subclinical hypothyroidism, while the exposed group in the current study were mothers with overt hypothyroidism. The results may not be directly comparable and the role of gestational exposure to L-T4 requires further study.

A clinical trial performed among thyroid peroxidase antibody positive (TPOAb+) mothers with normal to elevated levels of thyroid-stimulating hormone found no difference in preterm birth and miscarriage rate between mothers with and without L-T4 treatment during pregnancy [21]. However, prospective studies reported a lower risk of adverse pregnancy outcomes in TPOAb+ mothers who received L-T4 treatment during pregnancy compared to those without treatment [18–20]. Nevertheless, given the differences in study populations regarding maternal thyroid condition and thyroid-stimulating hormone levels, the results of preterm birth outcomes cannot be directly compared with ours.

To our knowledge, no previous study focused on investigating the association of maternal L-T4 use during pregnancy with the risk of both birth and neurodevelopmental outcomes in children. Only one placebo-controlled randomized trial was specifically designed to examine the efficacy of L-T4 treatment in pregnant women with subclinical hypothyroidism/hypothyroxinemia [15]. Psychological tests were conducted on their children at the age of five and the trial found no significant difference in neurodevelopmental outcomes between children of mothers with and without treatment. Two other clinical trials were carried out in pregnant women and they suggested a null association between maternal exposure to L-T4 and cognitive function in offspring [16, 17]. These trials partially support our findings that there was no change in risk of ADHD or ASD in children born from mothers exposed to L-T4 during pregnancy when compared to children of euthyroid mothers. Notably, our study has a longer mean follow-up time for the children compared to previous clinical trials. Previous trials started L-T4 treatment at a mean of 17 gestational weeks [15, 17],, or a median of 13 gestational weeks [16]. In our study, the L-T4 treatments commenced at a median of 18 gestational weeks. The observed null estimate for the neurodevelopmental outcomes may be explained by a substantial portion of L-T4 users who commenced their treatment at a late stage during pregnancy, at which L-T4 has minimal effect on the fetus compared with its effect during the first trimester. The effect of timing of L-T4 treatment during pregnancy requires further studies.

Our sensitivity analysis suggested that offspring sex does not influence the overall conclusion from the result. Though no significant association was observed between children with gestational L-T4 mothers and euthyroid mothers, the estimates of SGA and neurodevelopmental disorders were higher in boys than in girls. This finding was consistent with previous studies regarding ADHD and ASD among girls and boys [47, 48].

### Strengths and limitations

The current study had several strengths. First, the study analyzed a large population-based sample available as electronic health records from CDARS, with highly accurate and reliable information on the mother-child link. In addition, automated dispensing and prescribing records were applied to identify exposures and neurodevelopmental outcomes, which are free of recall bias. Second, our study involved longitudinal data and investigated both birth and neurodevelopmental outcomes associated with significant morbidity and mortality. Third, several sensitivity analyses suggested that the results from the main analysis were robust and reliable.

However, there were limitations. First, CDARS only contains medical records from publicly funded hospitals but not private hospitals and medical practitioners. In this study, we captured all birth events that occurred in public hospitals, which account for about 66% of births in Hong Kong. Though the remaining 34% in private healthcare were not covered in the current study, it is unlikely to affect the interpretation of our results as all comparisons were conducted in patients using HA services. In addition, the public sector is the main provider of medical care for neurodevelopmental disorders in Hong Kong. As children diagnosed with ADHD or ASD usually require long-term monitoring and treatment, they usually utilize health services provided by the public sector. Thus, they are most likely to be captured in this study. Second, exposure to L-T4 is defined by prescribing and dispensing records. Poor medication adherence could have biased the results towards the null. Nevertheless, we identified those with at least two L-T4 prescribing records to ameliorate this potential bias. Third, in Hong Kong, subclinical hypothyroidism (SCH) is not recommended to be treated. In this study, mothers who received L-T4 treatment were clinically diagnosed with hypothyroidism and were involved in routine clinical care. The interpretation of this study may not apply to individuals with SCH. Nevertheless, this study aimed to estimate the effect of gestational L-T4 treatment on offspring. Fourth, we could not tease apart the effects of hypothyroidism (including under-treated hypothyroidism) or gestational L-T4 treatment on offspring preterm birth as we did not account for thyroid hormone levels in the analysis. We recommend future research to adopt thyroid hormone lab test information and address the issues regarding disease severity and all forms of thyroid dysfunction. Fifth, though we applied different cut-offs considering the severity of preterm birth, we were not able to differentiate between spontaneous or medically indicated preterm birth. Sixth, given the nature of observational designs, all sources of confounding cannot be fully ruled out. We did not adjust the socioeconomic status of mothers in the cohort such as smoking or alcohol. To address potentially measured and unmeasured confounding, this study adopted additional sensitivity and subgroup analysis to help rule out some but not all sources of confounding to provide complementary evidence.

### Clinical implications and recommendations

There have been frequent updates in the guidelines of the American Thyroid Association (ATA) for L-T4 use in pregnant women in recent years. In 2011, ATA accepted thyroid-stimulating hormone of 2.5 mIU/L as the upper limit of the normal level in the first trimester [4], while similar patterns were observed in the association of L-T4 treatment during pregnancy and offspring birth and neurodevelopmental outcomes before and after this change in clinical practice. Current recommendations from ATA suggest L-T4 treatment should be based upon both measurements of thyroid function and thyroid peroxidase antibody status [2]. Our study supports this cautious decision since we found gestational L-T4 treatment was associated with an increased risk of preterm birth. Though the observed increased risk may also be due to hypothyroid disease itself, the use of L-T4 during pregnancy cannot reduce the risk of preterm birth sufficiently. Nevertheless, our results support gestational L-T4 treatment without identifying an increased risk of SGA, ADHD, or ASD. Importantly, the severity of hypothyroidism may also potentially influence the association and other major adverse outcomes may occur due to unstable maternal thyroid status if the treatment is abruptly stopped [49]. Clinicians and pregnant women need to have individual discussions about their gestational L-T4 treatment.

## Conclusions

The present study suggested that gestational exposure to L-T4 was associated with an increased risk of preterm birth but not SGA, ADHD, or ASD. Though we cannot completely exclude the possible effect of gestational L-T4 treatment on offspring preterm birth, such risk might be confounded by the underlying maternal thyroid disease itself. Decision-making about L-T4 use in pregnancy remains important and requires an assessment of the risks and benefits in the context of both mothers and children.

## Supplementary Information

**Additional file 1: Figure S1.** Pregnancy period and comparison groups’ identification. **Figure S2.** Cumulative incidence of ADHD and ASD by maternal L-T4 exposure. **Figure S3.** Box plot of the days between last L-T4 prescription and LMP of pre-pregnancy users. **Figure S4.** Box plot of cumulative dose among the gestational users during pregnancy. **Figure S5.** Box plot of length of time the mothers use L-T4 before pregnancy.

**Additional file 2: Table S1.** ICD-9-CM codes applied for covariates. **Table S2.** Covariates balance for comparison between gestational L-T4 users and euthyroid control. **Table S3.** Percentage of gestational users who started L-T4 treatment at different trimesters. **Table S4.** Covariates balance for the comparison between gestational users and pre-pregnancy users. **Table S5.** Sensitivity analysis by comparing gestational L-T4 users to pre-pregnancy. **Table S6.** Sensitivity analysis by excluding mothers exposed to psychotropic or antiepileptic medications before or during pregnancy. **Table S7.** Subgroup analysis by stratifying offspring sex. **Table S8.** Sensitivity analysis by using different SGA definitions. **Table S9.** Sensitivity analysis by different cut-offs for preterm birth. **Table S10.** Sensitivity analysis for the risk of offspring ADHD by restricting to children born before the year 2014. **Table S11.** Comparison between gestational L-T4 users before and after year 2011. **Table S12.** Interaction analysis of before and after year 2011 on the estimates in the comparison between gestational L-T4 users and euthyroid control. **Table S13.** Post-hoc analysis adjusted cumulative dose of L-T4 during pregnancy. **Table S14.** Post-hoc analysis adjusted length of time the mothers using L-T4 before pregnancy.

## Data Availability

The datasets analyzed in the current study are not publicly available due to the nature of ethical restriction. All anonymized data supporting the findings of this study are available within the article and its additional files on reasonable request.

## Abbreviations

ADHD: Attention-deficit/hyperactivity disorder
ASD: Autism spectrum disorder
L-T4: Levothyroxine
CDARS: Clinical Data Analysis and Reporting System
HA: Hospital Authority
BNF: British National Formulary
RAI: Radioactive iodine
LMP: Last menstrual period
ICD-9-CM: International Classification of Diseases, 9th Revision, Clinical Modification
SGA: Small for gestational age
SD: Standard deviation
SMD: Standardized mean differences
PS: Propensity score
wOR: Weighted odds ratios
wHR: Weighted hazard ratios
CI: Confidence interval
TPOAb+: Thyroid peroxidase antibody positive
ATA: American Thyroid Association
