# Supplementary material for: Association of maternal levothyroxine use during pregnancy with offspring birth and neurodevelopmental outcomes: a population-based cohort study
Source: BMC Med. 2022 Nov 8;20:390. doi: 10.1186/s12916-022-02586-9 (PMC9641874; doi:10.1186/s12916-022-02586-9)
Supplement: Supplementary file 1 — Additional file 1: Figure S1. Pregnancy period and comparison groups’ identification. Figure S2. Cumulative incidence of ADHD and ASD by maternal L-T4 exposure. Figure S3. Box plot of the days between last L-T4 prescription and LMP of pre-pregnancy users. Figure S4. Box plot of cumulative dose among the gestational users during pregnancy. Figure S5. Box plot of length of time the mothers use L-T4 before pregnancy. [file 12916_2022_2586_MOESM1_ESM.docx]

**Fig. S1**

**
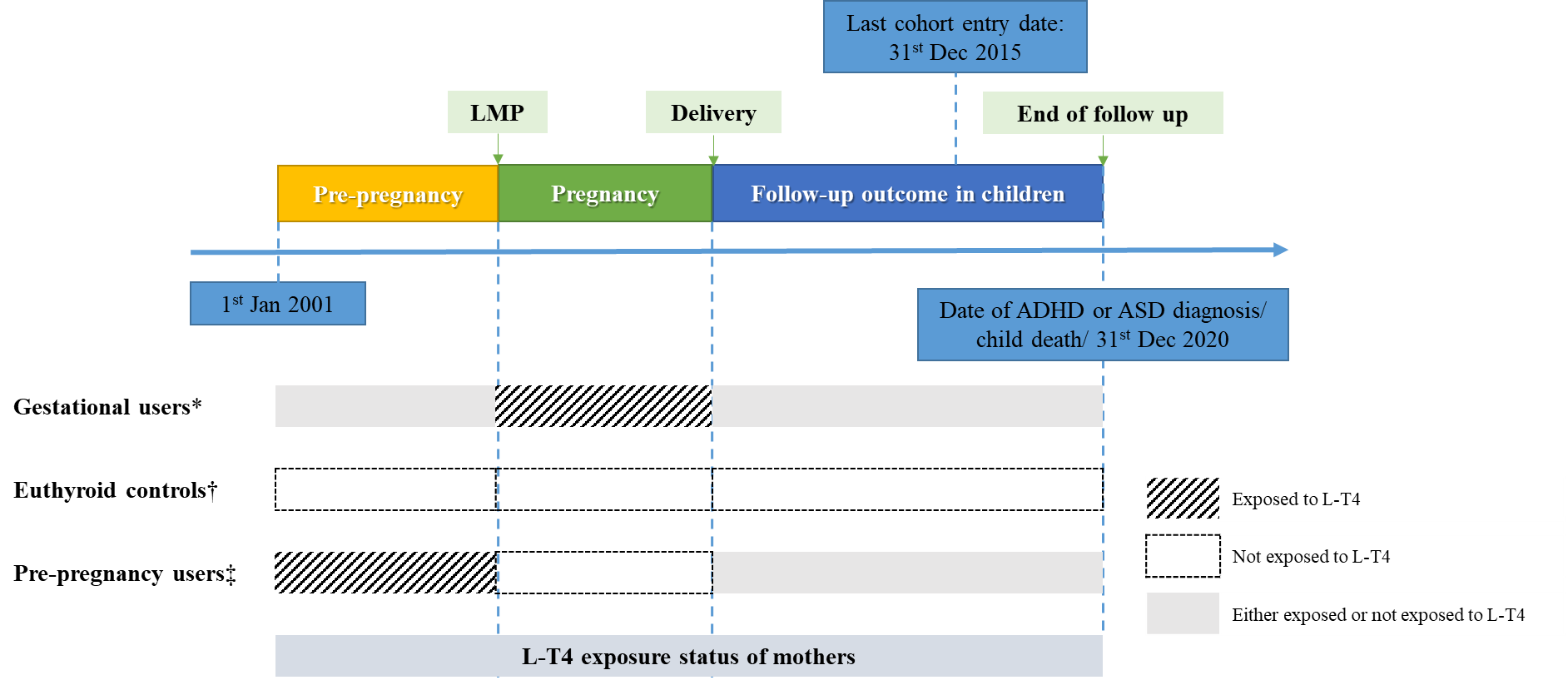
**

Pregnancy period and comparison groups’ identification.

The pregnancy period was defined as the period between LMP and the date of delivery. Comparison groups were classified based on maternal L-T4 exposure status.

*gestational users: mothers exposed to L-T4 during pregnancy

†euthyroid control: mothers have never been exposed to L-T4 and had no history of thyroid-related diagnosis

‡pre-pregnancy users: mothers exposed to L-T4 before pregnancy but stopped treatment when pregnant

LMP: last menstrual period; L-T4: levothyroxine

**Fig. S2**

**
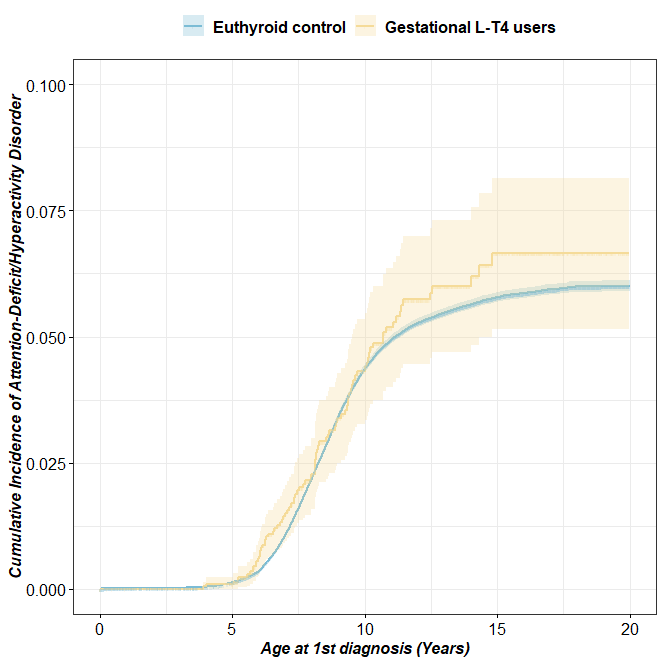

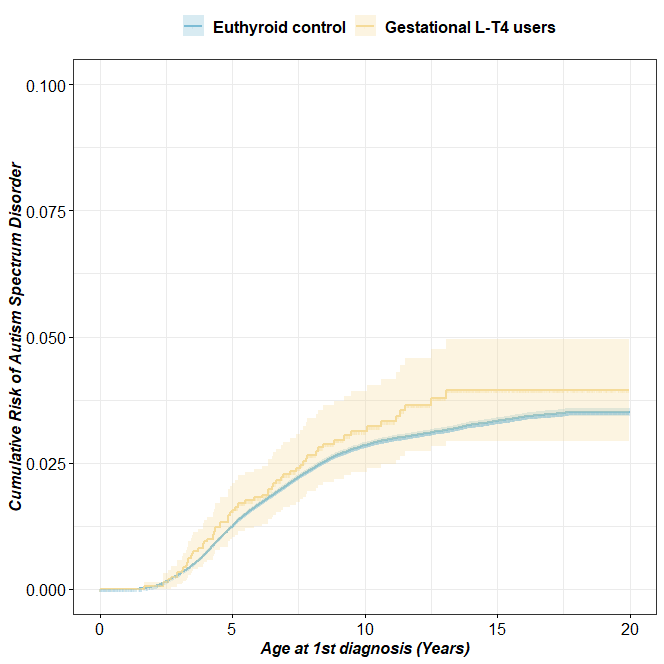
**

Cumulative incidence of attention-deficit/hyperactivity disorder and autism spectrum disorder by maternal L-T4 exposure

Children born from gestational L-T4 users had a similar probability of ADHD and ASD diagnosis when compared to children born from euthyroid control mothers.

**Fig. S3**

**
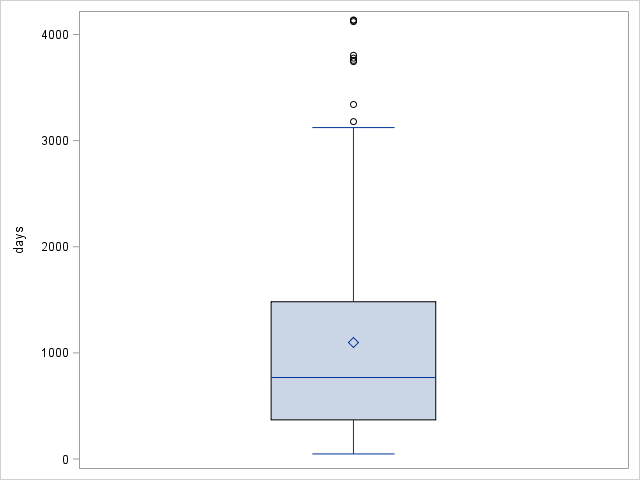
**

|  | **Median** | **Lower quartile** | **Upper quartile** |
| --- | --- | --- | --- |
| **Days** | 768 | 369 | 1482 |

Box plot of the days between last L-T4 prescription and last menstrual period of pre-pregnancy users

**Fig. S4**

|  | **Lower quartile** | **Median** | **Upper quartile** |
| --- | --- | --- | --- |
| **Cumulative L-T4 dose during pregnancy (mcg)** | 4900 | 13150 | 22900 |

**
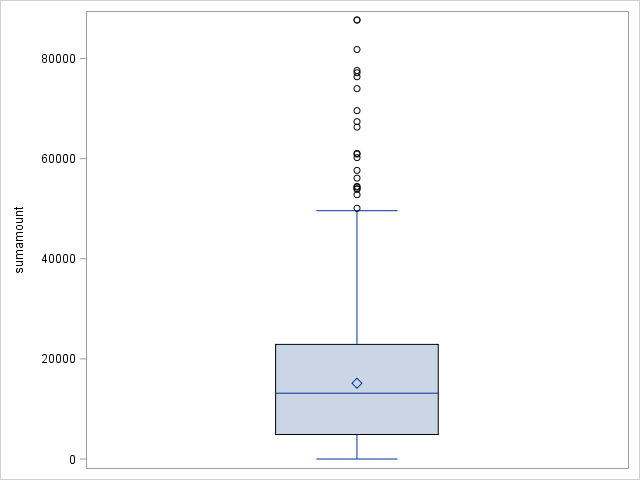
**

Box plot of cumulative L-T4 dose among the gestational users during pregnancy

**
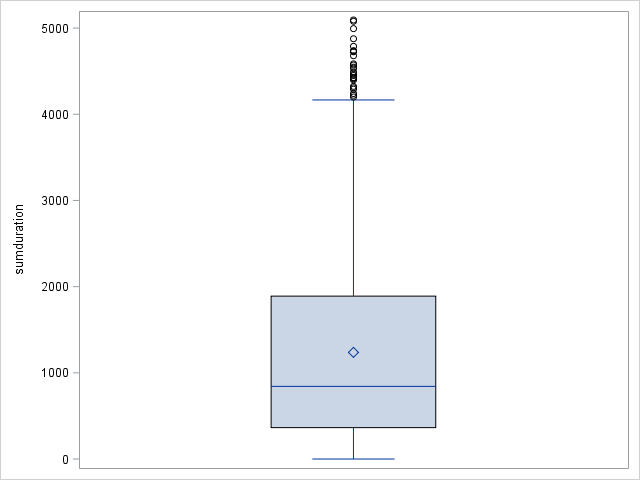
Fig. S5**

|  | **Lower quartile** | **Median** | **Upper quartile** |
| --- | --- | --- | --- |
| **Cumulative time exposed to L-T4 before last menstrual period (days)** | 364 | 843 | 1890 |

Box plot of length of time that the gestational exposed mothers used L-T4 before pregnancy
