# Supplementary material for: Association of maternal levothyroxine use during pregnancy with offspring birth and neurodevelopmental outcomes: a population-based cohort study
Source: BMC Med. 2022 Nov 8;20:390. doi: 10.1186/s12916-022-02586-9 (PMC9641874; doi:10.1186/s12916-022-02586-9)
Supplement: Supplementary file 2 — Additional file 2: Table S1. ICD-9-CM codes applied for covariates. Table S2. Covariates balance for comparison between gestational L-T4 users and euthyroid control. Table S3. Percentage of gestational users who started L-T4 treatment at different trimesters. Table S4. Covariates balance for the comparison between gestational users and pre-pregnancy users. Table S5. Sensitivity analysis by comparing gestational L-T4 users to pre-pregnancy. Table S6. Sensitivity analysis by excluding mothers exposed to psychotropic or antiepileptic medications before or during pregnancy. Table S7. Subgroup analysis by stratifying offspring sex. Table S8. Sensitivity analysis by using different SGA definitions. Table S9. Sensitivity analysis by different cut-offs for preterm birth. Table S10. Sensitivity analysis for the risk of offspring ADHD by restricting to children born before the year 2014. Table S11. Comparison between gestational L-T4 users before and after year 2011. Table S12. Interaction analysis of before and after year 2011 on the estimates in the comparison between gestational L-T4 users and euthyroid control. Table S13. Post-hoc analysis adjusted cumulative dose of L-T4 during pregnancy. Table S14. Post-hoc analysis adjusted length of time the mothers using L-T4 before pregnancy. [file 12916_2022_2586_MOESM2_ESM.docx]

**Table S1 International Classification of Diseases,** **9^th^ Revision, Clinical Modification codes applied for covariates**

| **Diagnosis** | **ICD-9-CM codes** |
| --- | --- |
| Hypertension | 401-415, 437.2 |
| Epilepsy | 345 |
| Psychiatric disorders | 290-319 |
| Gestational diabetes | 648.83 |
| Pre-existing diabetes | 250 |

**Table S2 Covariate balance before and after propensity score weighting for comparison between gestational L-T4 users and euthyroid control**

|  | **Gestational L-T4 users vs Euthyroid control SMD (%)** | |
| --- | --- | --- |
|  | **Before PS weighting** | **After PS weighting** |
| **Sample size** | Exposed group: 2,125  Unexposed group: 398,909 | Exposed group: 2,124  Unexposed group: 395,824 |
| **Maternal age at delivery** | 50.6 | 0.4 |
| **Parity** | 5.8 | 0.2 |
| **Calendar year at delivery** | 3.4 | 0.1 |
| **Maternal underlying conditions:** |  |  |
| ***Gestational diabetes*** | 14.9 | 0.2 |
| ***Pre-existing diabetes*** | 9.2 | 1.4 |
| ***Hypertension*** | 15 | 0.6 |
| ***Epilepsy*** | 8.2 | 1.5 |
| ***Psychiatric disorders*** | 9 | 0.6 |
| **Birth hospitals:** |  |  |
| **1** | 12.1 | 0.1 |
| **2** | 9.2 | 0.1 |
| **3** | 3.7 | 0.1 |
| **4** | 5.3 | 0.1 |
| **5** | 1.3 | 0.4 |
| **6** | 20.7 | 0 |
| **7** | 3.4 | 0 |
| **8** | 4.7 | 0.2 |
| **9** | 3.7 | 0.1 |

SMD: standardized mean difference; PS: propensity score

**Table S3 Percentage of gestational users who started L-T4 treatment at different trimesters**

| **Timing of start L-T4 treatment during pregnancy** | **Number of mothers** | **Percentage (%)** |
| --- | --- | --- |
| 1^st^ trimester | 973 | 45.79 |
| 2^nd^ trimester | 595 | 28.00 |
| 3^rd^ trimester | 557 | 26.21 |

Gestational L-T4 users started L-T4 treatment at a median of 18 gestational weeks. 1^st^ trimester: 0 to 12 weeks after last menstrual period (LMP); 2^nd^ trimester: 13 to 26 weeks after LMP; 3^rd^ trimester: 27 weeks after LMP till delivery.

**Table S4 Covariate balance before and after propensity score weighting for the comparison between gestational users and pre-pregnancy users**

|  | **SMD (%)** | |
| --- | --- | --- |
|  | **Gestational users vs Pre-pregnancy users** | |
|  | **Before PS weighting** | **After PS weighting** |
| **Sample size** | Exposed: 2,125  Unexposed: 173 | Exposed: 1,289  Unexposed: 172 |
| **Maternal age at delivery** | 35 | 1.3 |
| **Parity** | 5.6 | 0.1 |
| **Calendar year at delivery** | 3.6 | 0.1 |
| **Maternal underlying conditions:** |  |  |
| ***Gestational diabetes*** | 5.3 | 0.9 |
| ***Pre-existing diabetes*** | 1.6 | 0.2 |
| ***Hypertension*** | 3.3 | 0.6 |
| ***Epilepsy*** | 2.6 | 0.2 |
| ***Psychiatric disorders*** | 12.4 | 0.7 |
| **Birth hospitals:** |  |  |
| **1** | 2.0 | 0.5 |
| **2** | 6.6 | 0.2 |
| **3** | 14.0 | 0.9 |
| **4** | 11.3 | 0.5 |
| **5** | 35.5 | 1.4 |
| **6** | 13.2 | 0.7 |
| **7** | 4.4 | 0.5 |
| **8** | 9.7 | 0.5 |
| **9** | 5.8 | 0.2 |

SMD: standardized mean difference; PS: propensity score

**Table S5 Sensitivity analysis by comparing gestational L-T4 users to pre-pregnancy**

| **Outcomes** | **N of cases in exposed (%)** | **N of cases in unexposed (%)** | **Crude** | | **PS-weighted *** | |
| --- | --- | --- | --- | --- | --- | --- |
| **Gestational L-T4 users (N = 2,125) vs Pre-pregnancy users (N = 173)** | | | | | | |
|  |  |  | **OR (95% CI)** | **P-value** | **OR (95% CI)** | **P-value** |
| **Preterm birth** | 256 (12.05) | 10 (5.78) | 2.23 (1.16, 4.28) | 0.02 | 2.16 (1.09, 4.25) | 0.03 |
| **SGA** | 41 (1.93) | 2 (1.16) | 1.68 (0.40, 7.01) | 0.48 | 1.05 (0.29, 3.79) | 0.94 |
|  |  |  | **HR (95% CI)** | **P-value** | **HR (95% CI)** | **P-value** |
| **ADHD** | 85 (4.00) | 5 (2.89) | 1.27 (0.51, 3.12) | 0.61 | 1.39 (0.50, 3.86) | 0.53 |
| **ASD** | 65 (3.06) | 7 (4.05) | 0.72 (0.33, 1.58) | 0.42 | 0.60 (0.23, 1.53) | 0.28 |
| ***** PS-weighted model adjusted for maternal age at delivery, birth year, birth hospital, parity, maternal underlying illness before delivery including pre-existing diabetes, gestational diabetes, epilepsy, hypertension, and psychiatric conditions. | | | | | | |

OR: odds ratio; HR: hazard ratio; PS: propensity score; vs: versus; ADHD: attention-deficit/ hyperactivity disorder; ASD: autism spectrum disorder; SGA: small for gestational age

**Table S6 Sensitivity analysis by excluding mothers exposed to psychotropic or antiepileptic medications before or during pregnancy**

| **Gestational L-T4 users (N = 1,928) vs Euthyroid control (N = 378,675)** | | | | | | |
| --- | --- | --- | --- | --- | --- | --- |
| **Outcomes** | **N of cases in exposed (%)** | **N of cases in unexposed (%)** | **Crude** | | **PS-weighted *** | |
|  |  |  | **OR (95% CI)** | **P-value** | **OR (95% CI)** | **P-value** |
| **Preterm birth** | 230 (11.93) | 31,264 (8.26) | 1.51 (1.31, 1.73) | <0.0001 | 1.26 (1.10, 1.45) | 0.001 |
| **SGA** | 36 (1.87) | 6,368 (1.68) | 1.11 (0.80, 1.55) | 0.53 | 1.07 (0.76, 1.49) | 0.71 |
|  |  |  | **HR (95% CI)** | **P-value** | **HR (95% CI)** | **P-value** |
| **ADHD** | 73 (3.79) | 13,647 (3.60) | 1.10 (0.87, 1.38) | 0.42 | 1.11 (0.87, 1.40) | 0.41 |
| **ASD** | 57 (2.96) | 9,916 (2.62) | 1.15 (0.89, 1.49) | 0.29 | 1.03 (0.78, 1.35) | 0.86 |
| ***** PS-weighted model adjusted for maternal age at delivery, birth year, birth hospital, parity, maternal underlying illness before delivery including pre-existing diabetes, gestational diabetes, epilepsy, hypertension, and psychiatric conditions. | | | | | | |

OR: odds ratio; HR: hazard ratio; PS: propensity score; vs: versus; ADHD: attention-deficit/ hyperactivity disorder; ASD: autism spectrum disorder; SGA: small for gestational age

**Table S7 Subgroup analysis by stratifying offspring sex**

| **Gestational L-T4 users vs Euthyroid control** | | | | | | |
| --- | --- | --- | --- | --- | --- | --- |
| **Outcomes** | **N of cases in exposed (%)** | **N of cases in unexposed (%)** | **Crude** | | **PS-weighted *** | |
| **Girls** | | | | | | |
|  |  |  | **OR (95% CI)** | **P-value** | **OR (95% CI)** | **P-value** |
| **Preterm birth** | 116 (11.78) | 14,960 (7.80) | 1.58 (1.30, 1.92) | <0.0001 | 1.23 (1.01, 1.50) | 0.04 |
| **SGA** | 15 (1.52) | 3,167 (1.65) | 0.84 (0.49, 1.42) | 0.51 | 0.92 (0.56, 1.54) | 0.76 |
|  |  |  | **HR (95% CI)** | **P-value** | **HR (95% CI)** | **P-value** |
| **ADHD** | 16 (1.62) | 3,489 (1.82) | 0.92 (0.56, 1.50) | 0.73 | 0.91 (0.56, 1.49) | 0.72 |
| **ASD** | 7 (0.71) | 1,670 (0.87) | 0.82 (0.39, 1.73) | 0.61 | 0.72 (0.34, 1.51) | 0.38 |
| **Boys** | | | | | | |
|  |  |  | **OR (95% CI)** | **P-value** | **OR (95% CI)** | **P-value** |
| **Preterm birth** | 140 (12.28) | 18,502 (8.93) | 1.43 (1.20, 1.71) | <0.0001 | 1.20 (1.00, 1.43) | 0.05 |
| **SGA** | 26 (2.28) | 3,679 (1.78) | 1.29 (0.87, 1.91) | 0.20 | 1.27 (0.86, 1.87) | 0.24 |
|  |  |  | **HR (95% CI)** | **P-value** | **HR (95% CI)** | **P-value** |
| **ADHD** | 69 (6.05) | 11,663 (5.63) | 1.14 (0.90, 1.45) | 0.26 | 1.13 (0.88, 1.44) | 0.35 |
| **ASD** | 58 (5.09) | 9,157 (4.42) | 1.18 (0.91, 1.53) | 0.20 | 1.03 (0.79, 1.35) | 0.81 |
| ***** PS-weighted model adjusted for maternal age at delivery, birth year, birth hospital, parity, maternal underlying illness before delivery including pre-existing diabetes, gestational diabetes, epilepsy, hypertension, and psychiatric conditions. | | | | | | |

OR: odds ratio; HR: hazard ratio; PS: propensity score; vs: versus; ADHD: attention-deficit/ hyperactivity disorder; ASD: autism spectrum disorder; SGA: small for gestational age

**Table S8 Sensitivity analysis by using different SGA definitions**

| **Gestational L-T4 users (N = 2,125) vs Euthyroid control (N = 398,909)** | | | | | | |
| --- | --- | --- | --- | --- | --- | --- |
| **Outcome definitions** | **N of cases in exposed (%)** | **N of cases in unexposed (%)** | **Crude** | | **PS-weighted *** | |
|  |  |  | **OR (95% CI)** | **P-value** | **OR (95% CI)** | **P-value** |
| **SGA (<mean - 2*SD)** | 41 (1.93) | 6,787 (1.70) | 1.14 (0.83, 1.55) | 0.42 | 1.08 (0.79, 1.48) | 0.64 |
| **SGA (<5%)** | 120 (5.65) | 19,609 (4.92) | 1.16 (0.96, 1.39) | 0.12 | 1.14 (0.94, 1.37) | 0.18 |
| **SGA (<3%)** | 70 (3.29) | 11,659 (2.92) | 1.13 (0.89, 1.44) | 0.31 | 1.10 (0.86, 1.39) | 0.45 |
| ***** PS-weighted model adjusted for maternal age at delivery, birth year, birth hospital, parity, maternal underlying illness before delivery including pre-existing diabetes, gestational diabetes, epilepsy, hypertension, and psychiatric conditions. | | | | | | |

OR: odds ratio; OR: odds ratio; PS: propensity score; SGA: small for gestational age

SGA (<mean - 2*SD): SGA defined as birth weight less than 2 standard deviations (SD) below the mean of the same gestational age

SGA (<3%): SGA defined as the lowest 3rd percentile of the gestational age-specific birth weight

SGA (<5%): SGA defined as the lowest 5th percentile of the gestational age-specific birth weight

**Table S9 Sensitivity analysis by different cut-offs for preterm birth**

| **Gestational L-T4 users (N = 2,125) vs Euthyroid control (N = 398,909)** | | | | | | | | |
| --- | --- | --- | --- | --- | --- | --- | --- | --- |
| **Preterm birth severity** | **N of cases in exposed (%)** | **N of cases in unexposed (%)** | **Crude** | | **PS-weighted *** | | **PS-weighted **** | |
|  |  |  | **OR (95% CI)** | **P-value** | **OR (95% CI)** | **P-value** | **OR (95% CI)** | **P-value** |
| **Moderate to late (<37 weeks)** | 256 (12.05) | 33,462 (8.39) | 1.50 (1.32, 1.71) | <0.0001 | 1.22 (1.07, 1.39) | 0.003 | 1.22 (1.07, 1.38) | 0.003 |
| **Extremely**  **(<28 weeks)** | 22 (1.04) | 1,667 (0.42) | 2.49 (1.63, 3.81) | <0.0001 | 1.94 (1.27, 2.96) | 0.002 | 1.94 (1.27, 2.96) | 0.002 |
| **Very**  **(<33 weeks)** | 73 (3.44) | 7,359 (1.84) | 1.89 (1.50, 2.39) | <0.0001 | 1.43 (1.14, 1.82) | 0.002 | 1.43 (1.13, 1.81) | 0.003 |
| ***** PS-weighted model adjusted for maternal age at delivery, birth year, birth hospital, parity, maternal underlying illness before delivery including pre-existing diabetes, gestational diabetes, epilepsy, hypertension, and psychiatric conditions.  ****** PS-weighted model adjusted for maternal age at delivery, birth year, birth hospital, parity, maternal underlying illness before delivery including pre-existing diabetes, gestational diabetes, epilepsy, hypertension, psychiatric conditions, and pre-eclampsia. | | | | | | | | |

OR: odds ratio; OR: odds ratio; PS: propensity score

Moderate to late (<37 weeks) preterm birth: children born less than 37 gestational weeks. This definition was applied in the main analysis.

Extremely (<28 weeks) preterm birth: children born less than 28 gestational weeks

Very (<33 weeks) preterm birth: children born less than 33 gestational weeks

**Table S10 Sensitivity analysis of maternal L-T4 exposure with risk of ADHD by restricting to children born before the year 2014**

| **Gestational L-T4 users (N = 1,658) vs Euthyroid control (N = 323,525)** | | | | | | |
| --- | --- | --- | --- | --- | --- | --- |
| **Outcome** | **N of cases in exposed (%)** | **N of cases in unexposed (%)** | **Crude** | | **PS-weighted *** | |
|  |  |  | **HR (95% CI)** | **P-value** | **HR (95% CI)** | **P-value** |
| **ADHD** | 82 (4.95) | 14,742 (4.56) | 1.10 (0.89, 1.37) | 0.38 | 1.10 (0.88, 1.38) | 0.39 |
| ***** PS-weighted model adjusted for maternal age at delivery, birth year, birth hospital, parity, maternal underlying illness before delivery including pre-existing diabetes, gestational diabetes, epilepsy, hypertension, and psychiatric conditions. | | | | | | |

HR: hazard ratio; PS: propensity score; vs: versus; ADHD: attention-deficit/ hyperactivity disorder

**Table S11 Comparison between gestational L-T4 users before and after year 2011**

| **Gestational L-T4 users vs Euthyroid control** | | | | | | |
| --- | --- | --- | --- | --- | --- | --- |
| **Outcomes** | **N of cases in exposed (%)** | **N of cases in unexposed (%)** | **Crude** | | **PS-weighted *** | |
| **Before year 2011** | | | | | | |
|  |  |  | **OR (95% CI)** | **P-value** | **OR (95% CI)** | **P-value** |
| **Preterm birth** | 139 (12.72) | 18,854 (8.74) | 1.53 (1.28, 1.82) | <0.0001 | 1.22 (1.02, 1.45) | 0.032 |
| **SGA** | 17 (1.56) | 3,789 (1.76) | 0.88 (0.55, 1.43) | 0.61 | 0.86 (0.53, 1.38) | 0.52 |
|  |  |  | **HR (95% CI)** | **P-value** | **HR (95% CI)** | **P-value** |
| **ADHD** | 59 (5.40) | 11,438 (5.30) | 1.01 (0.79, 1.31) | 0.37 | 1.04 (0.81, 1.35) | 0.74 |
| **ASD** | 38 (3.48) | 6,063 (2.81) | 1.24 (0.90, 1.70) | 0.19 | 1.13 (0.82, 1.58) | 0.45 |
| **After year 2011** | | | | | | |
|  |  |  | **OR (95% CI)** | **P-value** | **OR (95% CI)** | **P-value** |
| **Preterm birth** | 101 (11.57) | 11,939 (7.99) | 1.51 (1.23, 1.86) | 0.0001 | 1.21 (0.98, 1.49) | 0.08 |
| **SGA** | 20 (2.29) | 2,419 (1.62) | 1.43 (0.92, 2.23) | 0.12 | 1.35 (0.87, 2.11) | 0.19 |
|  |  |  | **HR (95% CI)** | **P-value** | **HR (95% CI)** | **P-value** |
| **ADHD** | 17 (1.95) | 2,218 (1.48) | 1.40 (0.87, 2.25) | 0.17 | 1.26 (0.76, 2.09) | 0.37 |
| **ASD** | 25 (2.86) | 3,379 (2.53) | 1.15 (0.78, 1.71) | 0.48 | 1.01 (0.67, 1.51) | 0.98 |
| ***** PS-weighted model adjusted for maternal age at delivery, birth year, birth hospital, parity, maternal underlying illness before delivery including pre-existing diabetes, gestational diabetes, epilepsy, hypertension, and psychiatric conditions. | | | | | | |

OR: odds ratio; HR: hazard ratio; PS: propensity score; vs: versus; ADHD: attention-deficit/ hyperactivity disorder; ASD: autism spectrum disorder; SGA: small for gestational age

**Table S12 Interaction analysis of before and after year 2011 on the estimates in the comparison between gestational L-T4 users and euthyroid control**

| **Interaction analysis** | |
| --- | --- |
| **Outcomes** | **P-value** |
| **Preterm birth** | 0.58 |
| **SGA** | 0.40 |
| **ADHD** | 0.20 |
| **ASD** | 0.20 |

Interaction analysis indicating the effect of before and after year 2011 on the estimates for each outcome. The comparison was between gestational L-T4 users and euthyroid control. ADHD: attention-deficit/ hyperactivity disorder; ASD: autism spectrum disorder; SGA: small for gestational age.

**Table S13 Post-hoc analysis adjusted cumulative dose of L-T4 during pregnancy**

| **Outcomes** | **N of cases in exposed (%)** | **N of cases in unexposed (%)** | **Crude** | | **PS-weighted *** | |
| --- | --- | --- | --- | --- | --- | --- |
| **Low cumulative dose (N = 1,046) vs Euthyroid control ((N = 398,909)** | | | | | | |
|  |  |  | **OR (95% CI)** | **P-value** | **OR (95% CI)** | **P-value** |
| **Preterm birth** | 126 (12.05) | 33,462 (8.39) | 1.49 (1.24, 1.81) | <.0001 | 1.21 (1.01, 1.46) | 0.04 |
| **SGA** | 21 (2.01) | 6,787 (1.70) | 1.18 (0.77, 1.83) | 0.44 | 1.09 (0.70, 1.69) | 0.72 |
|  |  |  | **HR (95% CI)** | **P-value** | **HR (95% CI)** | **P-value** |
| **ADHD** | 36 (3.44) | 15,152 (3.80) | 1.00 (0.72, 1.39) | 1.00 | 0.98 (0.70, 1.38) | 0.92 |
| **ASD** | 32 (3.06) | 10,827 (2.71) | 1.18 (0.83, 1.67) | 0.35 | 1.00 (0.70, 1.43) | 1.00 |
| **High cumulative dose (N = 1,079) vs Euthyroid control (N = 398,909)** | | | | | | |
|  |  |  | **OR (95% CI)** | **P-value** | **OR (95% CI)** | **P-value** |
| **Preterm birth** | 130 (12.05) | 33,462 (8.39) | 1.50 (1.25, 1.81) | <.0001 | 1.23 (1.02, 1.48) | 0.029 |
| **SGA** | 20 (1.85) | 6,787 (1.70) | 1.10 (0.70, 1.70) | 0.69 | 1.07 (0.69, 1.67) | 0.76 |
|  |  |  | **HR (95% CI)** | **P-value** | **HR (95% CI)** | **P-value** |
| **ADHD** | 49 (4.54) | 15,152 (3.80) | 1.19 (0.90, 1.57) | 0.23 | 1.19 (0.89, 1.59) | 0.24 |
| **ASD** | 33 (3.06) | 10,827 (2.71) | 1.13 (0.80, 1.59) | 0.50 | 1.01 (0.71, 1.45) | 0.96 |
| **Low cumulative dose (N = 1,046) vs High cumulative dose (N = 1,079)** | | | | | | |
|  |  |  | **OR (95% CI)** | **P-value** | **OR (95% CI)** | **P-value** |
| **Preterm birth** | 126 (12.05) | 130 (12.05) | 1.00 (0.77, 1.23) | 1.00 | 0.99 (0.77, 1.29) | 0.96 |
| **SGA** | 21 (2.01) | 20 (1.85) | 1.09 (0.59, 2.01) | 0.80 | 1.24 (0.65, 2.35) | 0.51 |
|  |  |  | **HR (95% CI)** | **P-value** | **HR (95% CI)** | **P-value** |
| **ADHD** | 36 (3.44) | 49 (4.54) | 0.84 (0.55, 1.30) | 0.44 | 0.81 (0.50, 1.31) | 0.39 |
| **ASD** | 32 (3.06) | 33 (3.06) | 1.05 (0.64, 1.70) | 0.85 | 0.89 (0.52, 1.52) | 0.68 |
| ***** PS-weighted model adjusted for maternal age at delivery, birth year, birth hospital, parity, maternal underlying illness before delivery including pre-existing diabetes, gestational diabetes, epilepsy, hypertension, and psychiatric conditions. | | | | | | |

**Table S14 Post-hoc analysis adjusted length of time the mothers using L-T4 before pregnancy**

| **Outcomes** | **N of cases in exposed (%)** | **N of cases in unexposed (%)** | **Crude** | | **PS-weighted *** | |
| --- | --- | --- | --- | --- | --- | --- |
| **Short cumulative days (N = 517) vs Euthyroid control (N = 398,909)** | | | | | | |
|  |  |  | **OR (95% CI)** | **P-value** | **OR (95% CI)** | **P-value** |
| **Preterm birth** | 55 (10.64) | 33,462 (8.39) | 1.30 (0.98, 1.72) | 0.07 | 1.14 (0.80, 1.50) | 0.37 |
| **SGA** | 7 (1.35) | 6,787 (1.70) | 0.79 (0.38, 1.67) | 0.54 | 0.72 (0.32, 1.60) | 0.41 |
|  |  |  | **HR (95% CI)** | **P-value** | **HR (95% CI)** | **P-value** |
| **ADHD** | 22 (4.26) | 15,152 (3.80) | 1.23 (0.91, 1.61) | 0.18 | 1.25 (0.93, 1.66) | 0.15 |
| **ASD** | 15 (2.90) | 10,827 (2.71) | 1.08 (0.65, 1.79) | 0.77 | 1.02 (0.62, 1.70) | 0.93 |
| **Long cumulative days (N = 1,608) vs Euthyroid control (N = 398,909)** | | | | | | |
|  |  |  | **OR (95% CI)** | **P-value** | **OR (95% CI)** | **P-value** |
| **Preterm birth** | 201 (12.50) | 33,462 (8.39) | 1.56 (1.35, 1.81) | <0.0001 | 1.24 (1.07, 1.44) | 0.004 |
| **SGA** | 34 (2.11) | 6,787 (1.70) | 1.25 (0.89, 1.76) | 0.20 | 1.18 (0.84, 1.66) | 0.35 |
|  |  |  | **HR (95% CI)** | **P-value** | **HR (95% CI)** | **P-value** |
| **ADHD** | 63 (3.86) | 15,152 (3.80) | 0.99 (0.81, 1.18) | 1.00 | 1.00 (0.75, 1.33) | 1.00 |
| **ASD** | 50 (3.11) | 10,827 (2.71) | 1.17 (0.89, 1.54) | 0.27 | 1.19 (0.91, 1.47) | 0.31 |
| **Short cumulative days (N = 517) vs Long cumulative days (N = 1,608)** | | | | | | |
|  |  |  | **OR (95% CI)** | **P-value** | **OR (95% CI)** | **P-value** |
| **Preterm birth** | 55 (10.64) | 201 (12.50) | 0.83 (0.61, 1.14) | 0.26 | 0.95 (0.69. 1.31) | 0.75 |
| **SGA** | 7 (1.35) | 34 (2.11) | 0.64 (0.28, 1.44) | 0.28 | 0.56 (0.25, 1.27) | 0.16 |
|  |  |  | **HR (95% CI)** | **P-value** | **HR (95% CI)** | **P-value** |
| **ADHD** | 22 (4.26) | 63 (3.86) | 1.14 (0.91, 1.37) | 0.17 | 1.16 (0.93, 1.39) | 0.15 |
| **ASD** | 15 (2.90) | 50 (3.11) | 0.91 (0.51, 1.63) | 0.76 | 1.07 (0.58, 1.97) | 0.83 |
| ***** PS-weighted model adjusted for maternal age at delivery, birth year, birth hospital, parity, maternal underlying illness before delivery including pre-existing diabetes, gestational diabetes, epilepsy, hypertension, and psychiatric conditions. | | | | | | |
